# Methyl 3-(4-chloro­phen­yl)-2-(1,3-dimethyl-2,5-dioxo-4-phenyl­imidazolidin-4-yl)-3-oxopropano­ate

**DOI:** 10.1107/S1600536811018733

**Published:** 2011-05-25

**Authors:** Dongxue Zhang, Cong Deng, Yan Yang

**Affiliations:** a Key Laboratory of Pesticide and Chemical Biology of the Ministry of Education, College of Chemistry, Central China Normal University, Wuhan 430079, People’s Republic of China

## Abstract

The title compound, C_21_H_19_ClN_2_O_5_, is a tetra­substituted hydantoin derivative which contains an imidazolidine-2,4-dione core. The dihedral angle between the aromatic rings is 64.53 (14)°. In the crystal, weak inter­molecular C—H⋯O hydrogen bonding is found. An intra­molecular C—H⋯O inter­action also occurs.

## Related literature

For the preparation of the title compound, see: Gao *et al.* (2010 ▶). 
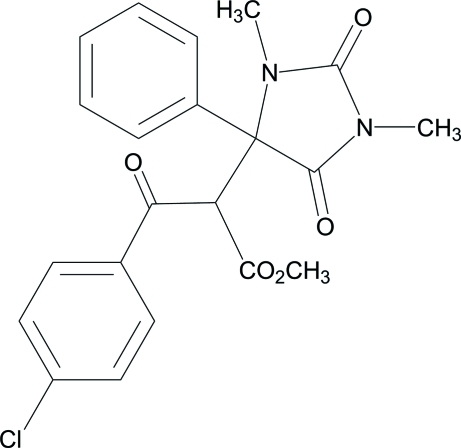


## Experimental

### 

#### Crystal data

C_21_H_19_ClN_2_O_5_
*M* *_r_* = 414.83
Monoclinic, 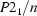

*a* = 11.4644 (11) Å
*b* = 12.0231 (12) Å
*c* = 15.1184 (15) Å
β = 101.731 (2)°
*V* = 2040.4 (3) Å^3^
*Z* = 4
Mo *K*α radiation
μ = 0.22 mm^−1^
*T* = 298 K
0.40 × 0.30 × 0.20 mm

#### Data collection

Bruker SMART CCD area-detector diffractometer
21013 measured reflections
4007 independent reflections
3573 reflections with *I* > 2σ(*I*)
*R*_int_ = 0.059

#### Refinement

*R*[*F* ^2^ > 2σ(*F* ^2^)] = 0.069
*wR*(*F* ^2^) = 0.164
*S* = 1.17
4007 reflections
265 parameters
H-atom parameters constrained
Δρ_max_ = 0.32 e Å^−3^
Δρ_min_ = −0.22 e Å^−3^

### 

Data collection: *SMART* (Bruker, 1997 ▶); cell refinement: *SAINT* (Bruker, 1999 ▶); data reduction: *SAINT*; program(s) used to solve structure: *SHELXS97* (Sheldrick, 2008 ▶); program(s) used to refine structure: *SHELXL97* (Sheldrick, 2008 ▶); molecular graphics: *SHELXTL* (Sheldrick, 2008 ▶); software used to prepare material for publication: *SHELXTL*.

## Supplementary Material

Crystal structure: contains datablocks I, global. DOI: 10.1107/S1600536811018733/nc2228sup1.cif

Structure factors: contains datablocks I. DOI: 10.1107/S1600536811018733/nc2228Isup2.hkl

Supplementary material file. DOI: 10.1107/S1600536811018733/nc2228Isup3.cml

Additional supplementary materials:  crystallographic information; 3D view; checkCIF report

## Figures and Tables

**Table 1:** Hydrogen-bond geometry (Å, °)

*D*—H⋯*A*	*D*—H	H⋯*A*	*D*⋯*A*	*D*—H⋯*A*
C3—H3⋯O3^i^	0.93	2.49	3.168 (3)	129
C10—H10*A*⋯O5^ii^	0.96	2.51	3.235 (4)	132
C15—H15*B*⋯O2	0.96	2.59	3.312 (4)	132

Symmetry codes: (i) 
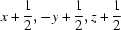
; (ii) 
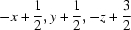
.

**Fig. 1.**
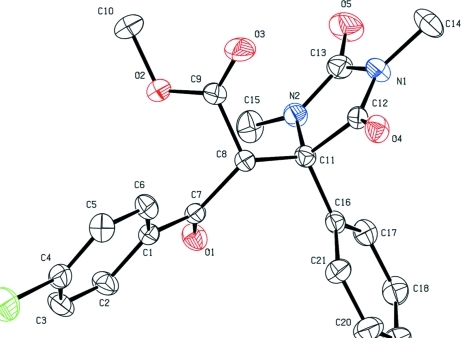
A view of (I), showing the atom-labelling scheme, with displacement ellipsoids drawn at the 30% probability level. H atoms omitted for clarity.

## supplementary crystallographic information

### Experimental

The title compound was synthesized according to the reported literature (Gao *et al.*, 2010). The crystal was grown by slow evaporation of the solvent at room temperature from a chloroform-methanol(1:1) solution of the title compound.

### Refinement

All H atoms were positioned in geometrically idealized positions and constrained to ride on their parent atoms, with C—H distances in the range of 0.93–0.98 Å and *U*_iso_(H) = 1.2*U*_eq_(C) (1.5 for methyl H atoms).

### Crystal data

**Table:**

C_21_H_19_ClN_2_O_5_	*F*(000) = 864
*M**_r_* = 414.83	*D*_x_ = 1.350 Mg m^−^^3^
Monoclinic, *P*2_1_/*n*	Mo *K*α radiation, λ = 0.71073 Å
Hall symbol: -P 2yn	Cell parameters from 6439 reflections
*a* = 11.4644 (11) Å	θ = 2.2–27.8°
*b* = 12.0231 (12) Å	µ = 0.22 mm^−^^1^
*c* = 15.1184 (15) Å	*T* = 298 K
β = 101.731 (2)°	Block, colourless
*V* = 2040.4 (3) Å^3^	0.40 × 0.30 × 0.20 mm
*Z* = 4	

### Data collection

**Table:**

Bruker SMART CCD area-detector diffractometer	3573 reflections with *I* > 2σ(*I*)
Radiation source: fine-focus sealed tube	*R*_int_ = 0.059
graphite	θ_max_ = 26.0°, θ_min_ = 2.0°
φ and ω scans	*h* = −14→14
21013 measured reflections	*k* = −14→14
4007 independent reflections	*l* = −18→18

### Refinement

**Table:**

Refinement on *F*^2^	Primary atom site location: structure-invariant direct methods
Least-squares matrix: full	Secondary atom site location: difference Fourier map
*R*[*F*^2^ > 2σ(*F*^2^)] = 0.069	Hydrogen site location: inferred from neighbouring sites
*wR*(*F*^2^) = 0.164	H-atom parameters constrained
*S* = 1.17	*w* = 1/[σ^2^(*F*_o_^2^) + (0.0623*P*)^2^ + 1.2007*P*] where *P* = (*F*_o_^2^ + 2*F*_c_^2^)/3
4007 reflections	(Δ/σ)_max_ = 0.001
265 parameters	Δρ_max_ = 0.32 e Å^−^^3^
0 restraints	Δρ_min_ = −0.22 e Å^−^^3^

### Special details

**Table:**

Geometry. All e.s.d.'s (except the e.s.d. in the dihedral angle between two l.s. planes) are estimated using the full covariance matrix. The cell e.s.d.'s are taken into account individually in the estimation of e.s.d.'s in distances, angles and torsion angles; correlations between e.s.d.'s in cell parameters are only used when they are defined by crystal symmetry. An approximate (isotropic) treatment of cell e.s.d.'s is used for estimating e.s.d.'s involving l.s. planes.
Refinement. Refinement of *F*^2^ against ALL reflections. The weighted *R*-factor *wR* and goodness of fit *S* are based on *F*^2^, conventional *R*-factors *R* are based on *F*, with *F* set to zero for negative *F*^2^. The threshold expression of *F*^2^ > σ(*F*^2^) is used only for calculating *R*-factors(gt) *etc*. and is not relevant to the choice of reflections for refinement. *R*-factors based on *F*^2^ are statistically about twice as large as those based on *F*, and *R*- factors based on ALL data will be even larger.

### Fractional atomic coordinates and isotropic or equivalent isotropic displacement parameters (Å^2^)

**Table:**

	*x*	*y*	*z*	*U*_iso_*/*U*_eq_	
C1	0.6491 (2)	0.2716 (2)	0.94021 (16)	0.0355 (5)	
C2	0.6948 (3)	0.3041 (2)	1.02851 (18)	0.0487 (7)	
H2	0.7238	0.2505	1.0718	0.058*	
C3	0.6979 (3)	0.4144 (3)	1.05292 (19)	0.0551 (8)	
H3	0.7286	0.4356	1.1122	0.066*	
C4	0.6548 (2)	0.4928 (2)	0.9885 (2)	0.0482 (7)	
C5	0.6083 (3)	0.4641 (2)	0.90104 (19)	0.0493 (7)	
H5	0.5786	0.5183	0.8585	0.059*	
C6	0.6058 (2)	0.3535 (2)	0.87671 (17)	0.0443 (6)	
H6	0.5750	0.3333	0.8172	0.053*	
C7	0.6478 (2)	0.1512 (2)	0.91874 (15)	0.0350 (5)	
C8	0.6010 (2)	0.1162 (2)	0.81994 (15)	0.0344 (5)	
H8	0.6337	0.1691	0.7821	0.041*	
C9	0.4663 (2)	0.1244 (2)	0.79297 (18)	0.0437 (6)	
C10	0.2854 (3)	0.1228 (4)	0.8447 (3)	0.0785 (11)	
H10A	0.2548	0.1872	0.8102	0.118*	
H10B	0.2578	0.1223	0.9006	0.118*	
H10C	0.2582	0.0569	0.8109	0.118*	
C11	0.6383 (2)	−0.0019 (2)	0.79438 (15)	0.0347 (5)	
C12	0.6180 (2)	−0.0061 (2)	0.69012 (16)	0.0378 (6)	
C13	0.5134 (2)	−0.1486 (2)	0.7368 (2)	0.0483 (7)	
C14	0.5159 (3)	−0.1377 (3)	0.5715 (2)	0.0702 (10)	
H14A	0.4433	−0.1012	0.5435	0.105*	
H14B	0.5033	−0.2166	0.5712	0.105*	
H14C	0.5771	−0.1207	0.5386	0.105*	
C15	0.5383 (3)	−0.1235 (3)	0.9003 (2)	0.0591 (8)	
H15A	0.5019	−0.1958	0.8957	0.089*	
H15B	0.4867	−0.0708	0.9207	0.089*	
H15C	0.6130	−0.1263	0.9426	0.089*	
C16	0.7700 (2)	−0.0284 (2)	0.82963 (15)	0.0352 (5)	
C17	0.8069 (3)	−0.1364 (2)	0.85344 (17)	0.0442 (6)	
H17	0.7505	−0.1925	0.8509	0.053*	
C18	0.9261 (3)	−0.1613 (3)	0.88070 (19)	0.0548 (7)	
H18	0.9493	−0.2339	0.8966	0.066*	
C19	1.0107 (3)	−0.0798 (3)	0.8845 (2)	0.0615 (8)	
H19	1.0910	−0.0964	0.9040	0.074*	
C20	0.9752 (3)	0.0270 (3)	0.8592 (2)	0.0607 (8)	
H20	1.0322	0.0823	0.8606	0.073*	
C21	0.8562 (2)	0.0526 (2)	0.83176 (19)	0.0457 (6)	
H21	0.8336	0.1250	0.8146	0.055*	
Cl1	0.66072 (9)	0.63209 (7)	1.01981 (7)	0.0773 (3)	
N1	0.5525 (2)	−0.0993 (2)	0.66416 (14)	0.0480 (6)	
N2	0.55847 (19)	−0.08964 (18)	0.81188 (14)	0.0425 (5)	
O1	0.68288 (17)	0.08220 (15)	0.97634 (11)	0.0474 (5)	
O2	0.41472 (16)	0.12565 (17)	0.86363 (13)	0.0524 (5)	
O3	0.41480 (19)	0.1265 (2)	0.71633 (14)	0.0767 (7)	
O4	0.65545 (17)	0.05911 (16)	0.64252 (12)	0.0486 (5)	
O5	0.4498 (2)	−0.22967 (19)	0.73042 (17)	0.0748 (7)	

### Atomic displacement parameters (Å^2^)

**Table:**

	*U*^11^	*U*^22^	*U*^33^	*U*^12^	*U*^13^	*U*^23^
C1	0.0306 (12)	0.0426 (13)	0.0341 (12)	−0.0007 (10)	0.0082 (9)	−0.0021 (10)
C2	0.0519 (16)	0.0500 (16)	0.0380 (14)	0.0056 (13)	−0.0052 (12)	−0.0034 (12)
C3	0.0582 (18)	0.0561 (17)	0.0433 (15)	0.0039 (14)	−0.0077 (13)	−0.0166 (13)
C4	0.0420 (14)	0.0442 (15)	0.0596 (17)	−0.0026 (12)	0.0135 (13)	−0.0134 (13)
C5	0.0577 (17)	0.0422 (15)	0.0504 (16)	0.0018 (13)	0.0165 (13)	0.0041 (12)
C6	0.0520 (15)	0.0484 (15)	0.0325 (13)	−0.0016 (12)	0.0086 (11)	−0.0028 (11)
C7	0.0290 (12)	0.0449 (13)	0.0312 (12)	0.0007 (10)	0.0064 (9)	−0.0012 (10)
C8	0.0341 (12)	0.0382 (13)	0.0310 (12)	−0.0004 (10)	0.0065 (9)	−0.0027 (10)
C9	0.0386 (14)	0.0498 (15)	0.0397 (14)	0.0030 (11)	0.0005 (11)	−0.0070 (12)
C10	0.0333 (16)	0.119 (3)	0.082 (2)	0.0004 (18)	0.0098 (15)	−0.023 (2)
C11	0.0357 (12)	0.0376 (13)	0.0316 (12)	−0.0044 (10)	0.0087 (10)	−0.0019 (10)
C12	0.0358 (13)	0.0432 (14)	0.0343 (13)	0.0058 (11)	0.0066 (10)	−0.0043 (11)
C13	0.0426 (15)	0.0441 (15)	0.0574 (17)	−0.0059 (12)	0.0084 (12)	−0.0077 (13)
C14	0.084 (2)	0.073 (2)	0.0471 (18)	−0.0079 (18)	−0.0021 (16)	−0.0221 (16)
C15	0.072 (2)	0.0542 (17)	0.0597 (19)	−0.0116 (15)	0.0324 (16)	0.0037 (14)
C16	0.0392 (13)	0.0381 (13)	0.0286 (11)	0.0018 (10)	0.0080 (10)	−0.0029 (10)
C17	0.0537 (16)	0.0414 (14)	0.0381 (14)	0.0002 (12)	0.0104 (12)	0.0021 (11)
C18	0.0620 (19)	0.0526 (17)	0.0472 (16)	0.0210 (15)	0.0047 (13)	0.0053 (13)
C19	0.0415 (16)	0.074 (2)	0.0642 (19)	0.0140 (15)	−0.0004 (14)	−0.0005 (16)
C20	0.0378 (15)	0.065 (2)	0.077 (2)	−0.0027 (14)	0.0069 (14)	−0.0041 (16)
C21	0.0388 (14)	0.0416 (14)	0.0568 (16)	0.0012 (11)	0.0102 (12)	0.0016 (12)
Cl1	0.0874 (7)	0.0451 (4)	0.0994 (7)	−0.0030 (4)	0.0187 (5)	−0.0212 (4)
N1	0.0512 (13)	0.0505 (13)	0.0394 (12)	−0.0044 (11)	0.0023 (10)	−0.0118 (10)
N2	0.0428 (12)	0.0443 (12)	0.0424 (12)	−0.0103 (9)	0.0132 (9)	−0.0039 (10)
O1	0.0588 (12)	0.0461 (10)	0.0353 (10)	0.0056 (9)	0.0046 (8)	0.0028 (8)
O2	0.0337 (10)	0.0734 (14)	0.0498 (11)	0.0006 (9)	0.0076 (8)	−0.0116 (10)
O3	0.0454 (12)	0.133 (2)	0.0455 (12)	0.0053 (13)	−0.0058 (9)	−0.0075 (13)
O4	0.0559 (11)	0.0557 (11)	0.0364 (10)	0.0022 (9)	0.0147 (8)	0.0041 (8)
O5	0.0732 (15)	0.0613 (14)	0.0883 (17)	−0.0323 (12)	0.0122 (13)	−0.0147 (12)

### Geometric parameters (Å, °)

**Table:**

C1—C2	1.387 (3)	C11—C12	1.547 (3)
C1—C6	1.394 (4)	C12—O4	1.201 (3)
C1—C7	1.483 (3)	C12—N1	1.361 (3)
C2—C3	1.375 (4)	C13—O5	1.209 (3)
C2—H2	0.9300	C13—N2	1.348 (3)
C3—C4	1.373 (4)	C13—N1	1.400 (4)
C3—H3	0.9300	C14—N1	1.453 (3)
C4—C5	1.366 (4)	C14—H14A	0.9600
C4—Cl1	1.738 (3)	C14—H14B	0.9600
C5—C6	1.378 (4)	C14—H14C	0.9600
C5—H5	0.9300	C15—N2	1.460 (3)
C6—H6	0.9300	C15—H15A	0.9600
C7—O1	1.211 (3)	C15—H15B	0.9600
C7—C8	1.540 (3)	C15—H15C	0.9600
C8—C9	1.518 (3)	C16—C21	1.384 (4)
C8—C11	1.554 (3)	C16—C17	1.390 (3)
C8—H8	0.9800	C17—C18	1.377 (4)
C9—O3	1.189 (3)	C17—H17	0.9300
C9—O2	1.322 (3)	C18—C19	1.372 (5)
C10—O2	1.452 (3)	C18—H18	0.9300
C10—H10A	0.9600	C19—C20	1.377 (5)
C10—H10B	0.9600	C19—H19	0.9300
C10—H10C	0.9600	C20—C21	1.378 (4)
C11—N2	1.456 (3)	C20—H20	0.9300
C11—C16	1.529 (3)	C21—H21	0.9300
			
C2—C1—C6	118.4 (2)	O4—C12—C11	126.2 (2)
C2—C1—C7	118.1 (2)	N1—C12—C11	106.3 (2)
C6—C1—C7	123.5 (2)	O5—C13—N2	127.8 (3)
C3—C2—C1	120.9 (3)	O5—C13—N1	124.2 (3)
C3—C2—H2	119.5	N2—C13—N1	108.0 (2)
C1—C2—H2	119.5	N1—C14—H14A	109.5
C4—C3—C2	119.1 (3)	N1—C14—H14B	109.5
C4—C3—H3	120.5	H14A—C14—H14B	109.5
C2—C3—H3	120.5	N1—C14—H14C	109.5
C5—C4—C3	121.8 (3)	H14A—C14—H14C	109.5
C5—C4—Cl1	119.5 (2)	H14B—C14—H14C	109.5
C3—C4—Cl1	118.7 (2)	N2—C15—H15A	109.5
C4—C5—C6	119.0 (3)	N2—C15—H15B	109.5
C4—C5—H5	120.5	H15A—C15—H15B	109.5
C6—C5—H5	120.5	N2—C15—H15C	109.5
C5—C6—C1	120.8 (2)	H15A—C15—H15C	109.5
C5—C6—H6	119.6	H15B—C15—H15C	109.5
C1—C6—H6	119.6	C21—C16—C17	118.3 (2)
O1—C7—C1	121.6 (2)	C21—C16—C11	120.7 (2)
O1—C7—C8	120.6 (2)	C17—C16—C11	120.8 (2)
C1—C7—C8	117.7 (2)	C18—C17—C16	120.8 (3)
C9—C8—C7	112.17 (19)	C18—C17—H17	119.6
C9—C8—C11	107.97 (19)	C16—C17—H17	119.6
C7—C8—C11	115.58 (19)	C19—C18—C17	120.5 (3)
C9—C8—H8	106.9	C19—C18—H18	119.8
C7—C8—H8	106.9	C17—C18—H18	119.8
C11—C8—H8	106.9	C18—C19—C20	119.2 (3)
O3—C9—O2	124.9 (3)	C18—C19—H19	120.4
O3—C9—C8	122.7 (2)	C20—C19—H19	120.4
O2—C9—C8	112.4 (2)	C19—C20—C21	120.7 (3)
O2—C10—H10A	109.5	C19—C20—H20	119.6
O2—C10—H10B	109.5	C21—C20—H20	119.6
H10A—C10—H10B	109.5	C20—C21—C16	120.5 (3)
O2—C10—H10C	109.5	C20—C21—H21	119.7
H10A—C10—H10C	109.5	C16—C21—H21	119.7
H10B—C10—H10C	109.5	C12—N1—C13	111.6 (2)
N2—C11—C16	113.5 (2)	C12—N1—C14	125.0 (3)
N2—C11—C12	101.05 (18)	C13—N1—C14	123.1 (2)
C16—C11—C12	106.35 (18)	C13—N2—C11	112.1 (2)
N2—C11—C8	113.72 (19)	C13—N2—C15	121.2 (2)
C16—C11—C8	113.95 (19)	C11—N2—C15	126.2 (2)
C12—C11—C8	106.94 (19)	C9—O2—C10	116.6 (2)
O4—C12—N1	127.4 (2)		
			
C6—C1—C2—C3	−0.2 (4)	N2—C11—C16—C21	−169.5 (2)
C7—C1—C2—C3	−179.6 (3)	C12—C11—C16—C21	80.4 (3)
C1—C2—C3—C4	0.0 (5)	C8—C11—C16—C21	−37.2 (3)
C2—C3—C4—C5	0.5 (5)	N2—C11—C16—C17	15.7 (3)
C2—C3—C4—Cl1	−179.2 (2)	C12—C11—C16—C17	−94.4 (3)
C3—C4—C5—C6	−0.8 (4)	C8—C11—C16—C17	148.0 (2)
Cl1—C4—C5—C6	179.0 (2)	C21—C16—C17—C18	1.7 (4)
C4—C5—C6—C1	0.6 (4)	C11—C16—C17—C18	176.6 (2)
C2—C1—C6—C5	−0.1 (4)	C16—C17—C18—C19	−0.2 (4)
C7—C1—C6—C5	179.2 (2)	C17—C18—C19—C20	−1.2 (5)
C2—C1—C7—O1	1.6 (4)	C18—C19—C20—C21	1.1 (5)
C6—C1—C7—O1	−177.7 (2)	C19—C20—C21—C16	0.3 (5)
C2—C1—C7—C8	−177.9 (2)	C17—C16—C21—C20	−1.7 (4)
C6—C1—C7—C8	2.8 (3)	C11—C16—C21—C20	−176.6 (3)
O1—C7—C8—C9	105.8 (3)	O4—C12—N1—C13	−173.5 (3)
C1—C7—C8—C9	−74.8 (3)	C11—C12—N1—C13	8.1 (3)
O1—C7—C8—C11	−18.6 (3)	O4—C12—N1—C14	0.5 (4)
C1—C7—C8—C11	160.9 (2)	C11—C12—N1—C14	−178.0 (3)
C7—C8—C9—O3	162.1 (3)	O5—C13—N1—C12	176.9 (3)
C11—C8—C9—O3	−69.4 (3)	N2—C13—N1—C12	−2.7 (3)
C7—C8—C9—O2	−19.6 (3)	O5—C13—N1—C14	2.9 (5)
C11—C8—C9—O2	108.9 (2)	N2—C13—N1—C14	−176.7 (3)
C9—C8—C11—N2	−39.6 (3)	O5—C13—N2—C11	176.0 (3)
C7—C8—C11—N2	86.9 (2)	N1—C13—N2—C11	−4.4 (3)
C9—C8—C11—C16	−171.7 (2)	O5—C13—N2—C15	3.3 (5)
C7—C8—C11—C16	−45.2 (3)	N1—C13—N2—C15	−177.1 (2)
C9—C8—C11—C12	71.1 (2)	C16—C11—N2—C13	−104.7 (2)
C7—C8—C11—C12	−162.42 (19)	C12—C11—N2—C13	8.7 (3)
N2—C11—C12—O4	171.7 (2)	C8—C11—N2—C13	122.9 (2)
C16—C11—C12—O4	−69.6 (3)	C16—C11—N2—C15	67.5 (3)
C8—C11—C12—O4	52.5 (3)	C12—C11—N2—C15	−179.1 (2)
N2—C11—C12—N1	−9.8 (2)	C8—C11—N2—C15	−64.9 (3)
C16—C11—C12—N1	108.9 (2)	O3—C9—O2—C10	3.8 (4)
C8—C11—C12—N1	−129.0 (2)	C8—C9—O2—C10	−174.4 (3)

### Hydrogen-bond geometry (Å, °)

**Table:**

*D*—H···*A*	*D*—H	H···*A*	*D*···*A*	*D*—H···*A*
C3—H3···O3^i^	0.93	2.49	3.168 (3)	129
C10—H10A···O5^ii^	0.96	2.51	3.235 (4)	132
C15—H15B···O2	0.96	2.59	3.312 (4)	132

Symmetry codes: (i) *x*+1/2, −*y*+1/2, *z*+1/2; (ii) −*x*+1/2, *y*+1/2, −*z*+3/2.
